# Deciphering the Labyrinthine System of the Immune Microenvironment in Recurrent Glioblastoma: Recent Original Advances and Lessons from Clinical Immunotherapeutic Approaches

**DOI:** 10.3390/cancers13246156

**Published:** 2021-12-07

**Authors:** Elena Anghileri, Monica Patanè, Natalia Di Ianni, Irene Sambruni, Martina Maffezzini, Micaela Milani, Luisa Maddaloni, Bianca Pollo, Marica Eoli, Serena Pellegatta

**Affiliations:** 1 Unit of Molecular Neuro-Oncology, Fondazione IRCCS Istituto Neurologico Carlo Besta, 20133 Milan, Italy; elena.anghileri@istituto-besta.it (E.A.); natalia.diianni@istituto-besta.it (N.D.I.); irene.sambruni@istituto-besta.it (I.S.); martina.maffezzini@istituto-besta.it (M.M.); micaela.milani@istituto-besta.it (M.M.); luisa.maddaloni@istituto-besta.it (L.M.); marica.eoli@istituto-besta.it (M.E.); 2 Unit of Neuropathology, Fondazione IRCCS Istituto Neurologico Carlo Besta, 20133 Milan, Italy; monica.patane@istituto-besta.it (M.P.); bianca.pollo@istituto-besta.it (B.P.); 3 Unit of Immunotherapy of Brain Tumors, Fondazione IRCCS Istituto Neurologico Carlo Besta, Via Celoria, 11, 20133 Milan, Italy

**Keywords:** glioblastoma, recurrence, tumor microenvironment, tumor infiltrating lymphocytes, dysfunction, immunotherapy

## Abstract

**Simple Summary:**

Active communication between GBM cells and tumor-infiltrating immune components contributes to establishing an immunosuppressive environment where T cells are scarce and exhausted. This condition is particularly exacerbated upon recurrence, which is almost inevitable for GBM patients. Immunotherapeutic approaches, including checkpoint inhibitors, have demonstrated limited efficacy and failed to prolong survival or recurrent GBM patients. Nevertheless, many studies have shown that T cell priming is possible in the GBM microenvironment, and that T cell exhaustion or dysfunction can be reprogrammed. We will revisit data from the literature and report original results obtained from recurrent GBM patients treated with dendritic cells to demonstrate the role of the microenvironment in predicting immunotherapy response and influencing decisions for personalized therapies.

**Abstract:**

The interpretation of the presence and function of immune infiltration in glioblastoma (GBM) is still debated. Over the years, GBM has been considered a cold tumor that is less infiltrated by effector cells and characterized by a high proportion of immunosuppressive innate immune cells, including GBM-associated microglia/macrophages (GAMs). In this context, the failure of checkpoint inhibitors, particularly in recurrent GBM (rGBM), caused us to look beyond the clinical results and consider the point of view of immune cells. The tumor microenvironment in rGBM can be particularly hostile, even when exposed to standard immunomodulatory therapies, and tumor-infiltrating lymphocytes (TILs), when present, are either dysfunctional or terminally exhausted. However, after checkpoint blockade therapy, it was possible to observe specific recruitment of adaptive immune cells and an efficient systemic immune response. In this review article, we attempt to address current knowledge regarding the tumor and immune microenvironment in rGBM. Furthermore, immunosuppression induced by GAMs and TIL dysfunction was revisited to account for genetic defects that can determine resistance to therapies and manipulate the immune microenvironment upon recurrence. Accordingly, we reevaluated the microenvironment of some of our rGBM patients treated with dendritic cell immunotherapy, with the goal of identifying predictive immune indicators of better treatment response.

## 1. Introduction

In 2002, Erick Holland described glioblastoma multiforme (glioblastoma, GBM) as “the most aggressive of the gliomas” and colloquially defined it as “the terminator” [1]. Since then, the standard therapies for treatment, including neurosurgery, radiotherapy, and chemotherapy with temozolomide (TMZ), also named the *Stupp regimen* [2,3], have not been updated. However, impressive advances in neurosurgical techniques, including fluorescence-guided resection with 5-aminolevulinic acid (5-ALA) or fluorescein [4,5], and the development of experimental therapies shaped by cellular, molecular, and genetic characterizations have modestly improved the prognosis of patients to a median of 12 to 15 months [2,6]. Nevertheless, GBM recurrence is almost inevitable, and there is no standard of care for these patients, who have an overall survival (OS) of 9 months [7,8].

Immunotherapeutic approaches are considered an important option for treating both primary and recurrent GBM (rGBM), in some cases disappointing, and in other cases ineffective. However, we believe that even in cases of failure, it is necessary to reflect on not only the survival of patients, which is considered the primary endpoint in the vast majority of the clinical studies, but also the immunological data to understand more thoroughly what the gaps in treatment are and how to address them. These considerations may help to improve the approaches that require the involvement of the immune system.

We cannot ignore that GBM can rely on an authentic and adequately organized ecosystem, named the tumor microenvironment, where non-neoplastic and neoplastic cells interact and communicate with each other. Moreover, GBM tumors can be infiltrated by immune cells, most of which are derived from the bone marrow, comprise mostly myeloid cells, and are involved in tumor progression, angiogenesis, resistance to therapies, and immunosuppression. Within the tumor microenvironment, there are also T cells (usually defined as tumor-infiltrating lymphocytes, TILs), which are critical effector cells that can recognize and potentially eliminate cancer cells. However, within a highly immunosuppressive environment, TILs can lose their ability to attack cancer cells and become dysfunctional [9].

This general overview of the tumor microenvironment can reflect the condition of both primary and rGBM. However, therapeutic interventions, including radiotherapy and chemotherapy, substantially affect and modify the tumor microenvironment [10]. Based on that, we will attempt to summarize all the data describing the influence of immune cells with respect to recurrent versus primary GBM in matched samples from the same patient. Many other solid cancers, including melanoma, pancreatic, and ovarian cancer, benefit from an immunosuppressive microenvironment during progression and metastatic spread [11]. The tumor microenvironment can interfere with T cell trafficking and function, posing a crucial obstacle to personalized therapies. In very recent studies, the microenvironment of ovarian cancer [12] and prostate cancer bone metastases [13] was dissected using single-cell sequencing technologies, demonstrating the presence of monocytes and immature macrophages or inflammatory monocytes and M2 macrophages, respectively. In both cases, these immunosuppressive components can influence the T cell composition and dysfunction.

This review will analyze data from the literature and describe our recent results stemming from the molecular and histological characterization of rGBM treated with immunotherapy to prove that microenvironment may not inevitably represent an obstacle for GBM treatment. The immune contexture, which characterizes density, composition, and functional state of immune cells within the microenvironment, could be considered a clear indicator of the immunotherapy resistance or efficacy, addressing clinical decisions for further personalized therapies. 

## 2. Changes in the Microenvironment upon Recurrence: Looking for a Common Functional Pattern for Immune-Targeted Therapy

GBM-associated microglia/macrophages (GAMs) are pivotal players implicated in immunosuppression and closely related to cancer progression and invasion within the tumor microenvironment. GAMs, the most critical and abundant cell type, represent up to one-half of the tumor mass, with a substantial difference between microglia (accounting for 15% of all GAMs) and monocyte- and bone marrow-derived GAMs (accounting for 85%) [14].

Microglia and macrophages were initially hard to distinguish, as common methods primarily rely on only surface markers or gene signatures. However, more recent studies using innovative technologies, including single-cell RNA sequencing and CITE-seq, have demonstrated that microglia and macrophages are two distinct subpopulations with unique transcriptional and phenotypical patterns. Furthermore, these advances have allowed researchers to better define the distribution and contribution of resident and infiltrating cells [15].

Resident microglia are localized in peritumoral areas. Some studies described microglia as being involved in tumor progression when polarized toward the M2 subset and correlating with a poor prognosis as CD163+ cells. Most recently, it became clear that microglia are predominant in newly diagnosed GBM, whereas they are outnumbered by monocyte-derived GAMs in rGBM, particularly in hypoxic areas.

Still very little is known about changes in the immune microenvironment in rGBM. The main issue limiting comparative studies between matched primary and recurrent specimens is that only 20–30% of GBMs are subject to a second resection after recurrence, limiting the availability of surgical material for subsequent comparative studies. Furthermore, as there is no standard of care after recurrence onset, patients consequently undergo different treatment protocols, that can induce variable and sometimes unpredictable changes in the composition of the immune components within the tumor microenvironment.

The differences in T cell infiltration between 11 matched TIL samples from primary GBM and rGBM were thoroughly investigated by Mohme and colleagues [16]. rGBM displayed a higher proportion of CD8+ TILs than did primary GBM. Furthermore, specific characteristics were measured to evaluate the memory status of TILs, and five different stages were identified based on the expression of CD45RA, CCR7, and CD28: T naïve, T central memory, T transitional memory, T effector memory, and T terminal effector cells. Interestingly, rGBM has a higher number of infiltrating T effector memory CD8+ T cells than does primary GBM, whereas TILs preferentially have a transitory memory status. An exhaustion signature was investigated on TILs from the same pairs of surgical samples. PD-1 was the most remarkably expressed exhaustion marker on TILs from both primary GBM and rGBM and was expressed by CD8+ and CD4+ TILs. Other markers, including KLRG1 and CD57, that have been correlated with impaired proliferation or terminal differentiation, were investigated and showed higher expression on CD4+ TILs than CD8+ TILs. Tim-3, one of the most common exhaustion markers, negatively regulates proinflammatory cytokine production in activated T cells and is expressed on both CD4+ and CD8+ TILs. Finally, CD8+ TILs coexpressing CD45RO and HLA-DR, which is defined as an activated memory status and indicates T cells that retain restricted repertoire clonality, were prevalent in rGBM [16]. These observations confirm the presence of T cell infiltration within the tumor microenvironment and indicate a significant difference in the tumor microenvironment between primary GBM and rGBM. Remarkably, TILs in rGBM are preferentially CD8+, and their immunophenotype seems to suggest that the TILs were previously in an activation state and could undergo memory formation. However, these data need to be examined carefully, as the authors themselves point out, as the data are open to interpretation. CD57 expression was found to be reduced on CD8+ TILs in rGBM. If CD57 is to be considered a marker for terminal differentiation of effector cells, this observation could confirm persistent effector activity. However, as CD57 was also identified as a marker indicating cytolytic CD8+ T cells, the low proportion of CD57− expressing CD8+ TILs can reflect a dysfunctional state [16,17].

A more recent study utilized mass cytometry (CyTOF) and evaluated a total of 30 markers to more comprehensively characterize the GBM immune microenvironment and cell–cell interactions within the microenvironment [18]. The T cell subset revealed an accumulation of exhausted T cells and regulatory T cells (Tregs) at levels higher in the tumor than in peripheral blood. Natural killer (NK) cells showed high infiltration ability, as supported by the increased numbers of cells with CXC chemokine receptor 3 (CXCR3) expression but low interferon (IFN)γ levels, indicating poor cytolytic activity. These analyses describe a weak T cell compartment characterized by impaired antitumor effector ability, and no differences were revealed between primary GBM and rGBM. Of note, as the authors claimed in the discussion, this study was performed on a limited number of specimens (13 primary GBM and only 3 rGBM samples). Although demanding, one recommendation is implementing the same analyses on matched specimens, with the goal of characterizing the evolution of the GBM immune microenvironment even under the pressure of standard and/or experimental treatments.

In another study from the Verhaak group, the immunological changes upon GBM relapse induced by standard treatment were investigated with an exome mutation data analysis conducted with 45 matched pairs of primary GBM and rGBM. The percentage of CD8+ TILs was significantly higher in rGBM than in matched primary GBM samples. Moreover, an increased proportion of CD8+ TILs was observed in correlation with hypermutation in primary GBM and rGBM [19].

The increased proportion of CD8+ cells in rGBM can be explained as an indication of immune recognition and an attempt at tumor eradication, but a subsequent dysfunctional state prevails due to prolonged antigen exposure and an immunosuppressive microenvironment at the tumor site [9]. However, this shift could also be attributed to a different composition of GAMs, which are considered to be the primary immune cells within the GBM immune microenvironment that exert an immunosuppressive role. Indeed, some studies agree that the GAM signature within the immune microenvironment of primary GBM is different from that of rGBM, in which the proportion of GAMs is decreased [20]. Resident GAMs, which are microglia, were predominant in primary GBM; however, they were outnumbered by infiltrating GAMs upon recurrence, particularly in hypoxic areas. A significant reduction in the monocyte gene signature within the microenvironment of rGBM compared with primary GBM was revealed, suggesting a depletion of circulating monocytes. In general, both immune components, CD8+ T cells and GAMs can contribute to the progression and invasion ability of GBM cells. CD8+ T cells, when primed, can exert selective pressure on tumor cells, promoting escape variants, as recently observed in the case of metastases [21]. GAMs are recruited by GBM cells and, once inside the tumor, they are exposed to immunosuppressive factors and are reprogrammed towards phenotypes that sustain GBM invasion. They produce many factors, including TGFb, causing the production of metalloproteinase (MMP)2 and MMP9, which disrupt the extracellular matrix and promote GBM invasion [22,23]. Other factors, such as CCL8, EGF, and stress-inducible protein 1 (STI1), produced by GAMs were observed as involved in GBM invasion [24,25,26]. 

Tumor microenvironment composition has been reported to be affected by IDH mutation status. 

IDH wild-type primary GBM and rGBM had differential patterns of infiltrating GAMs across the different molecular subtypes identified by Verhaak and colleagues [19]. In particular, in a rGBM transitioning to the mesenchymal subtype, the proportion of M2-polarized macrophages was higher than that in primary GBM, despite the overall reduction in monocyte infiltration [19].

Similar observations were noted from a more recent study based on a single-cell characterization of the immune microenvironment of primary GBM and rGBM. It was confirmed that the impaired infiltration of monocytes was compensated by an increase in macrophages derived from microglia. This shift upon recurrence was considered a potential effect induced by therapies, including radiotherapy, that can directly compromise the persistence and expansion of microglia within the tumor microenvironment.

The infiltration and presence of macrophages is negatively correlated with CD8+ T cells owing to the ability of CD163+ CD68+ cells to suppress the effector activity of TILs. Furthermore, myeloid-derived suppressor cells (MDSCs), which are particularly abundant in the GBM microenvironment, can act directly and indirectly on CD8+ T cells by suppressing their activation, effector function, and migration ability [27].

This scenario has to be revised in cases of rGBM carrying an IDH mutation (IDH1 MUTANT astrocytoma according to 2021 WHO classification [28]), which is defined as a driver in gliomas, owing to the critical role of IDH in influencing the functional state of GAMs [29,30,31]. Furthermore, essential differences in the immune microenvironment composition were revealed by comparing IDH wild-type and IDH-mutant gliomas with innovative technologies such as CyTOF analysis and single-cell sequencing.

IDH-mutant gliomas are characterized by the prevalence of microglia and a low number of other immune cells, in particular T cells, in the tumor microenvironment. Notably, GAMs in IDH-mutant GBM comprise a smaller percentage but exert more proinflammatory activities than do GAMs from IDH-WT GBM, and microglia are reported to be the principal source of this proinflammatory milieu. Proinflammatory microglia within the tumor microenvironment of IDH-mutant GBM from untreated patients were also detected in treated patients with IDH-mutant GBM, correlating the general proinflammatory status of these GBMs with prolonged survival [31]. More recently, Friedrich and colleagues have shown that R-2-hydroxyglutarate in IDH1-mutant gliomas could induce infiltrating macrophages toward an immunosuppressive state [29], as investigated in a GL261 glioma model overexpressing mutant IDH [29]. These data illuminate another mechanism that can be exploited for new therapies.

## 3. “GBM Is Not an Irreversibly Cold Tumor”: A Lesson from Checkpoint Blockade Therapy

In an elegant review, Jackson, Choi, and Lim carefully analyzed the interaction between GBM and immune cells and introduced a new resistance classification to immunotherapy based on intrinsic and adaptive mechanisms [32]. Four categories of resistance stem from melding of the immunoediting process (the *‘Three Es’* model [33]) with the clinical definition of ‘hot’ and ‘cold’ cancers (i.e., T-cell-inflamed and non-T-cell-inflamed tumors); the latter is exclusively based on the response of patients to checkpoint inhibitors. Hot tumors, such as melanoma, show a response to checkpoint blockade: 50% efficacy in treated patients and an induced long-lasting response in 75% of patients. GBM is considered completely the opposite of a hot tumor: it is characterized by high intrinsic and high adaptive resistance, and in some cases has been defined as an immunological and hostile desert [34]. In this context, immunotherapy often fails to provide a clinical benefit, with only 10% of treated patients showing improved survival. One glaring example arises from the phase 3 clinical trials of Rindopepimut or CDX-110 for newly diagnosed GBM based on a vaccine targeting EGFRvIII; the treatment consists of a peptide conjugated to keyhole limpet hemocyanin (KLH) [35]. The treatment failed to increase patient survival, whereas a robust but clinically unrelated humoral response to Rindopepimut was detected. A cellular immune-related response would have provided information about the contribution of innate and/or adaptive effector cells, even considering the administration of TMZ in combination with Rindopepimut. Increasing evidence supports the critical involvement of TMZ in influencing the adaptive immune response, limiting the activation of the antitumor immune response and generating a memory status [36,37,38,39,40].

Another unexpected negative clinical result arose from a clinical study involving rGBM patients treated with anti-PD-1 therapy (NCT02017717 [41]). Checkpoint blockade therapy has revolutionized the treatment of patients diagnosed with advanced melanoma [42] and was hailed in 2013 as a breakthrough for cancer therapy [43].

The first study attesting the activity and, consequently, efficacy of anti-PD-1 therapy in a preclinical glioma model was described in 2013. Improved survival was observed in GL261 glioma-bearing mice treated with anti-PD-1 therapy combined with radiotherapy and was correlated with higher infiltration of cytotoxic T cells (identified as CD8+ IFNγ and TNFα double-positive) and a lower frequency of Tregs (characterized as CD4+ FoxP3+) compared with those in the single modality arm (anti-PD-1 therapy) [44]. Anti-PD-1, as a single agent or in combination with CTLA-4 blockade, significantly improved survival more than that mediated by anti-PD-L1 and anti-PD-L2 blockade. As in a previous study, improved survival was correlated with an increase in the effector activity of NK cells and CD8+ T cells. In parallel, the number of immunosuppressive cells within the microenvironment and draining lymph nodes significantly decreased [45].

In more recent studies, GL261 glioma-bearing mice were treated with anti-PD-1 in combination with anti-TIM-3 blockade and focal radiation, showing better survival than untreated and monotherapy-treated mice. Improved survival correlated with increased tumor infiltration of active and memory T cells [46]. Anti-PD-1 therapy was also efficient in prolonging the survival of GL261 glioma-bearing mice, particularly when combined with anti-T cell immunoglobulin and ITIM domain (TIGIT) therapy [47], where TIGIT is an immune checkpoint receptor that plays a critical role in cancer immunity [48]. The positive effect observed on survival was likely induced by an increased effector function of T cells and a decreased suppressive effect of Tregs [47]. Substantial evidence obtained in glioma models treated with checkpoint inhibitors might prove beneficial in GBM patients. However, the clinical study CheckMate-143 (identifier: NCT02017717), which involves the treatment of rGBM patients with nivolumab (anti-PD-1 therapy), was not successful, as nivolumab failed to exhibit clinical benefit [41]. Based on the positive data in advanced melanoma patients, and considering the efficacy observed in preclinical glioma models, the lack of clinical benefits observed in patients with rGBM was disappointing. Nivolumab, as a single therapeutic modality, versus bevacizumab did not meet the primary endpoint of OS. Improved survival was observed in rGBM patients with a methylated O6-methylguanine-DNA methyltransferase (MGMT) promoter and no baseline corticosteroid use, suggesting that the administration of steroids negatively impacts the immune response and consequently abrogates the function and activation of specific antitumor T cells [41,49]. The clinical responses to anti-PD-1 therapy are rare but relevant from an immunological point of view. Indeed, some independent studies have observed implications in specific immune responses. Cloughesy and colleagues noted that improved patient survival correlated with intratumoral infiltration of T cells sustained by a systemic immune response [50]. In a companion study, rGBM patients treated with anti-PD-1 therapy exhibited modulation of the tumor microenvironment characterized by increased *CXCL10*, *CCL4,* and *CCL3L1* transcript expression, representing specific chemoattractant signals involved in the intratumoral recruitment of adaptive immune cells.

Consequently, there was increased immune cell infiltration, including conventional CD4+ T cells, NK cells, and CD8+ T cells expressing CD69 and HLA-DR, indicating previous T cell activation [51]. Furthermore, a higher T-cell receptor (TCR) clonal diversity was revealed among TILs in the treated patients, and similar results were reported in both studies. In particular, the overall number of TCRα and TCRβ clones was higher after treatment with anti-PD-1 therapy and significantly correlated with prolonged patient survival [51]. In a retrospective study of a cohort of rGBM patients treated with anti-PD-1 therapy, specific genetic alterations were observed to potentially affect the patient’s responsiveness to anti-PD-1 therapy, thus manipulating the immune microenvironment [52]. The conclusions of this study supported the benefits of anti-PD-1 therapy, confirming prolonged OS in responsive patients. Notably, GBM samples from non-responsive patients were enriched for PTEN mutations and characterized by high levels of infiltrating and resident innate immune cells, including macrophages, microglia, and neutrophils, all of which were likely involved in establishing an immunosuppressive microenvironment and thus hindering the tumor infiltration of cytotoxic T cells. The spatial organization of the tumor microenvironment is influenced by the PTEN status, PTEN-mutant GBM appeared to be highly resistant to T cell infiltration, which calls back to a previous study where GBM cells with wild-type PTEN were more susceptible to recognition and elimination by tumor-specific T cells than those enriched for PTEN mutation [53]. In conclusion, specific genetic alterations can predict a GBM-resistant subtype and affect the responsiveness of the tumor to anti-PD-1 therapy and other immunotherapeutic approaches. Most importantly, genetic features may delineate the evolution of GBM cells under selective pressure in response to immunotherapy [52,53].

Recently, we described a young patient enrolled in the CheckMate 143 study who was exceptionally responsive to treatment [54]. This patient, who is still alive without further progression 83 months after the second surgery, has a hypermutator phenotype associated with germinal mutations of the mismatch repair (MMR) genes consistent with a diagnosis of Lynch syndrome. No PTEN mutation was detected. The primary GBM specimen was characterized as “warm” tumor tissue, but the rGBM tissue shifted toward a “hot” tumor after standard radiochemotherapy. In particular, strong infiltration of CD4+ FoxP3- T cells was detected in the recurrent tumor, whereas CD8+ T cells were differentially distributed between the center and the periphery of the primary specimen. These genetic and immunological characteristics significantly contribute to the treatment response; this can lead to a durable clinical benefit sustained by a robust systemic adaptive immune response during anti-PD-1 therapy [54].

More recently, the focus shifted toward delineating a direct effect of checkpoint blockade on GAMs, which have already been identified as the main immunosuppressive components within the immune microenvironment. In a preclinical study, mice genetically modified to eliminate CD8+ T cells were treated with PD-1 blockade. The absence of CD8+ T cells in glioma-bearing mice did not prevent the efficacy of anti-PD-1 therapy, for which the major target was represented by GAMs expressing PD-1. The treatment benefit was attributable to a significant decrease in these immunosuppressive components, whereas residual GAMs within the tumor microenvironment were enriched in the M1 antitumor proinflammatory phenotype, supporting the notion that anti-PD-1 therapy might exert a direct effect in regulating immunosuppression [55]. A preclinical study based on dendritic cell (DC)-based immunotherapy reported a direct involvement of myeloid cells infiltrating the microenvironment in suppressing TIL functionality [56]. In glioma-bearing mice, DC vaccinations were efficient in eliciting a local antitumor T cell response. However, TILs were inactivated via a PD-1/PD-L1 signaling mechanism triggered by myeloid cells. Indeed, PD-L1 expression was upregulated not only on cancer cells but also in myeloid cells within the microenvironment, supporting that these immune components can directly suppress and inhibit the functionality of TILs. Subsequent treatment with anti-PD-1 and CSF-1R blockade increased the infiltration of DC-induced T cells and, subsequently, their local expansion and activation [56].

Of particular interest, a symbiotic circuit was described as activated in PTEN-null GBM, where lysyl oxidase (LOX), a potent attractant of macrophages, was overexpressed. A high density of infiltrating macrophages is involved in sustaining cancer cell survival and activating angiogenesis [57]. The symbiotic interaction between macrophages and GBM cells was correlated with poor survival of GBM patients, and it may be assumed that this condition is consistent with the study by Zhao and colleagues, which stated that PTEN-mutant GBM is intrinsically resistant and does not respond to anti-PD-1 therapy [52].

Based on all these observations, we can conclude that T cells infiltrating the tumor microenvironment of GBMs are tumor-specific and can retain the ability to recognize and potentially eliminate tumor cells. However, TILs continuously exposed to suppressive and inhibitory signals from components of the immune microenvironment, such as GAMs, are exhausted. This condition should denote a hypofunctional but reversible state of these T cells. However, it was observed that T cells in a late dysfunctional state are not reprogrammable and undergo terminal differentiation [58]. Notably, epigenetic changes can be imprinted within TILs and compromise their reinvigoration. Furthermore, recovered exhausted T cells can retain irreversible damage at the transcriptional level that prevents optimal recovery despite antigen removal and resolution of chronic infections, as extensively demonstrated in a series of recent studies published in *Nature Immunology* [59,60,61,62].

## 4. Immunotherapy Is Unsuccessful When Exhausted TILs Are Not Reinvigorated

Exhaustion, also referred to as dysfunction, can be defined as a transcriptional program in T cells that elicits a hyporesponsive state. The presence of dysfunctional TILs within the tumor microenvironment can be considered an indicator of their ability to recognize and potentially target cancer cells. Indeed, a specialized subset of tumor-reactive TILs can be identified and specifically characterized based on specific surface markers and a molecular signature of critical factors involved in T cell exhaustion [63]. A gradient of dysfunction was described for TILs within the tumor microenvironment, supporting the inevitable but gradual differentiation of T cells. During their progressive differentiation toward late dysfunctionality, TILs lose their effector activity and become unable to fight cancer cells. However, in these conditions, TILs can acquire a novel and different function. In melanoma and non-small-cell lung cancer, it was possible to identify the coexistence of different subsets of TILs: not only proliferative and tumor-reactive CD8+ TILs but also a subset of dysfunctional CD8+ T cells that express and secrete CXCL13, leading to B cell recruitment and the formation of tertiary lymphoid structures at tumor sites; these tertiary lymphoid structures are considered to be a good prognostic factor in some solid cancers [63,64,65]. In light of this gradual differentiation program, we can potentially identify a specific time window within which personalized immunotherapy can work effectively.

CD8+ TILs are more abundant upon recurrence, as reported by multiple groups of scientists. These TILs are thought to be exhausted under the influence of a highly immunosuppressive microenvironment; however, there is no specific evidence regarding the dysfunctional state or the presence of a gradient of dysfunctional CD8+ T cells infiltrating rGBM. This characterization could be particularly informative in the case of treatment with checkpoint inhibitors and with active immunotherapy, including DC-based immunotherapy, whose activity requires the presence of a tumor-reactive subset of TILs or TILs that are amenable to reinvigoration. Evidence stemming from a clinical study of a group of patients affected by primary GBM treated with DC vaccination supports this phenomenon [66]. Therefore, T cell infiltration in GBM specimens was retrospectively investigated by immunohistochemistry (IHC), allowing the classification of the enrolled patients into two subgroups based on the presence of CD8+ TILs expressing PD-1. Counts of this subset revealed the prognostic significance of CD8+ TILs. In particular, a high PD-1+/CD8+ ratio was correlated with an increased estimated risk of death and, consequently, a poor prognosis, with a median progression-free survival (PFS) of 4.3 months and OS of 20.07 months. By contrast, patients with a low PD-1+/CD8+ ratio exhibited a median PFS of 11.2 months and a median OS of 60.9 months [66].

We can assume that upon recurrence, the proportion of CD8+ TILs is higher than that in primary GBM, predominantly in an exhaustion state, as TILs have already undergone prolonged exposure to tumor antigens and an immunosuppressive microenvironment. Over the years, we have gained more experience in treating primary GBM and rGBM patients with DC-based immunotherapy [39,67,68]. In addition, we have optimized immunological monitoring to characterize the peripheral immune system, and in some cases, we have confirmed a correlation between systemic and local immune responses [67].

In our (now closed) clinical study NCT04002804 (DENDR2), rGBM patients were treated with DC vaccination with TMZ as an adjuvant [39]. Four of twelve patients reached the survival endpoint of 9 months but showed no sign of immune response activation. A small subgroup of DENDR2 patients received DC vaccinations without TMZ and, alternatively, tetanus toxoid preconditioning as an adjuvant. In this cohort of patients, we observed significantly prolonged survival in five out of eight patients (OS > 9 months), with one patient still alive at the end of the evaluation period (OS > 68 months). Notably, this improved prognosis was correlated with the specific and robust activation of the peripheral immune response, which is characterized by a strong increase in CD8+ T cells sustained by CD4+ T cells and memory status generation [39]. These results have allowed us to reach important conclusions: first, TMZ administration as an adjuvant of DC vaccination prevents and suppresses the activation of specific antitumor immunity. This evidence is also supported by results from the DENDR1 (NCT04801147) clinical study, where TMZ negatively impacts CD8+ T cells and memory status generation [67]. Second, tetanus toxoid preconditioning and a lack of TMZ administration showed a positive effect in activating the antitumor immune response and improving survival. In these cases, we did not characterize the immune microenvironment, but we can hypothesize that the expected effects of TMZ in modulating the tumor microenvironment are not effective in rGBM patients and thus negatively impact the local and peripheral immune response.

## 5. IDH Mutation and Tumor Microenvironment Influence the Outcome of DENDR2 Patients

In light of the very recent advances in the characterization of the immune microenvironment at a single-cell resolution, we selected two DENDR2 patients, one with PFS < 9 months and one with PFS > 9 months, from whom paraffin-embedded and frozen surgical material was available and evaluated the immune infiltration and molecular pattern of the microenvironment by IHC and real-time PCR, respectively, to identify potential predictive indicators of immunotherapy response. We also considered investigating tissues from two IDH1-mutant rGBM patients treated with DC vaccination in combination with TMZ, owing to the differences in terms of the immune microenvironment compared to IDH1 wild-type GBM. Clinical characteristics of these patients are summarized in the Supplementary Data File.

**Figure 1:**
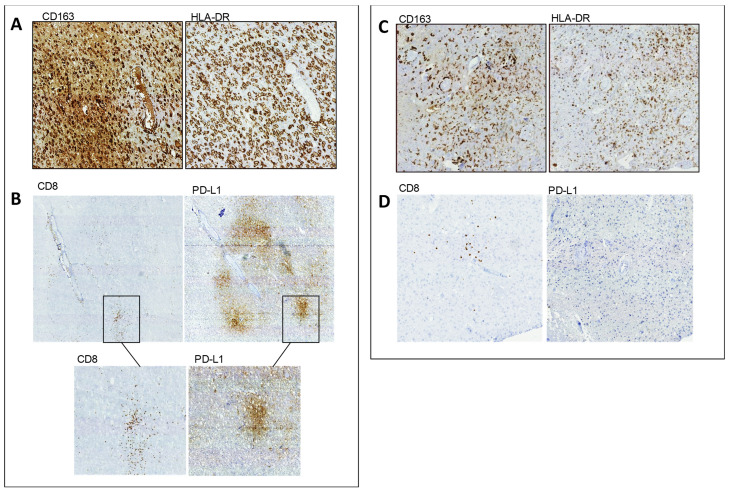
IHC performed on FFPE specimens of Pt#1 and Pt#3. In IDH1 wt rGBM (Pt#1), (**A**) adjacent representative sections show a high frequency of GAMs expressing CD163 (left) and HLA-DR(right). (**B**) the distribution of CD8+ T cells (left) is clustered near the blood vessels and within the tumor mass around the tumor cells, and the same areas of the adjacent sections express high levels of PD-L1. The black rectangle identifies intensely infiltrated CD8+ T cells and the corresponding area where tumor cells overexpress PD-L1. In IDH1-mutant (mut) rGBM (Pt#3), (**C**) adjacent representative sections show low frequency of GAMs expressing CD163 (left) and HLA-DR (right), with a prevalence of small size cells. (**D**) CD8+ TILs (left) are rare, and, when present, the distribution is scattered. PD-L-1 (right) expression is not detected.

A semiquantitative evaluation of immune cells within the microenvironment was performed by IHC (Table S1), revealing a relevant difference between the two subgroups of rGBM patients. In particular, in IDH1 wild-type rGBM (Pt#1 and Pt#2, Supplementary Data), we confirmed the presence of CD8+ T cells, which were not detected in IDH1-mutant rGBM (Pt#3 and Pt#4, Supplementary Data), and a higher proportion of CD163+ HLA-DR+ cells (Figure 1).

**Figure 2:**
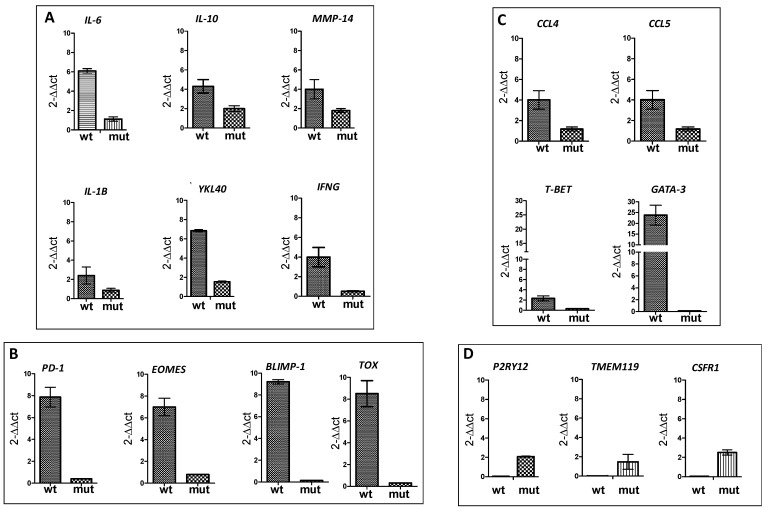
RT-PCR performed on frozen specimens of Pt#1 and Pt#3. (**A**) Relative expression of *IL-6*, *IL-10*, *MMP14*, and *IL-1B* is higher in wt rGBM than IDH1-mutant rGBM, indicating a prevalence of the M2-GAM subtype. *YKL40*, implicated in the regulation of GAM infiltration and migration, is upregulated in wt rGBM. *IFNG* expression is detected in wt rGBM, likely produced by CD8+ TILs. (**B**) Genes related to a terminal dysfunction of TILs are found in wt rGBM, where CD8+ TILs are abundant. *PD-1*, *EOMES*, *BLIMP-1*, and *TOX* indicate a terminal differentiation of infiltrating T cells. (**C**) *CCL4* and *CCL5* genes involved in TIL recruitment are upregulated in Pt#1. *T-BET* and *GATA3* genes indicating the Th subtype show a prevalence of Th2 phenotype supporting an immunosuppressive microenvironment. (**D**) A molecular signature related to microglia is upregulated in IDH1-mutant rGBM, including *P2RY12* and *TMEM119*, described as coexpressed in microglia.

A subsequent molecular characterization was performed on the rGBM specimens from two patients (Pt#1 IDH1 wt and Pt#3 IDH1-mutant rGBM) (Supplementary Methods and Table S2). Upregulation of M2 macrophage-dependent genes and the lack of expression of microglia-related genes revealed an immunosuppressive microenvironment of IDH1 wild-type DENDR2 patients, who had a worse prognosis. We observed upregulated IL6, IL-10, IL-1b, and MMP4 expression (Figure 2A). Notably, in one specimen, the expression of YKL40, a gene related to a mesenchymal phenotype [68] and involved in the modulation of macrophage-induced immunosuppression [69], was upregulated (Figure 2A). This observation, together with a remarkable infiltration of CD8+ TILs, is consistent with a recent report describing the contribution of infiltrating macrophages to inducing the mesenchymal transition of GBM cells. In turn, macrophages are induced to express a mesenchymal program preferentially associated with the presence of CD8+ T cells characterized by a cytotoxic program [68]. Therefore, we can predict that CD8+ TILs in a dysfunctional state caused the failure of immunotherapy in this DENDR2 case, as confirmed by the upregulation of genes related to late dysfunction, including *PD1,* BLIMP1 encoded by *PRDM1*, *EOMES,* and *TOX* (Figure 2B). Notably, the presence of dysfunctional CD8+ TILs can be assumed by the presence of PD-L1-expressing cells within the tumor microenvironment, as observed by IHC. This was likely induced by high expression levels of *IFN*γ (Figure 2A), which is already known to be involved in upregulating PD-L1 expression, thus triggering a mechanism of immune escape [70]. In addition, the upregulation of *CCL4* and *CCL5* (Figure 2C) expression can support the presence of a chemotactic gradient involved in CD8+ T cell recruitment. We also evaluated the expression of *T-BET* and *GATA3* (Figure 2C), the specific transcription factors related to the Th1 vs. Th2 phenotype, respectively, and their association with antitumor vs. protumor activity. We confirmed a strong upregulation of *GATA3,* indicating the prevalence of the Th2 phenotype and establishing a strong immunosuppressive microenvironment. 

In the IDH1-mutant rGBM patient, genes related to M2 macrophages were not expressed, whereas genes identifying microglia were significantly expressed. In particular, we investigated the expression of *CSF1R*, *P2RY12*, and *TMEM119*, with *TMEM119* (Figure 2D) exclusively expressed by microglia and usually coexpressed with *P2RY12* [71]. In addition, the prevalence of microglia in the IDH1-mutant rGBM sample was also confirmed by the presence of CD163+ HLA-DR+ cells, which are characterized by their small size. 

**Figure 3:**
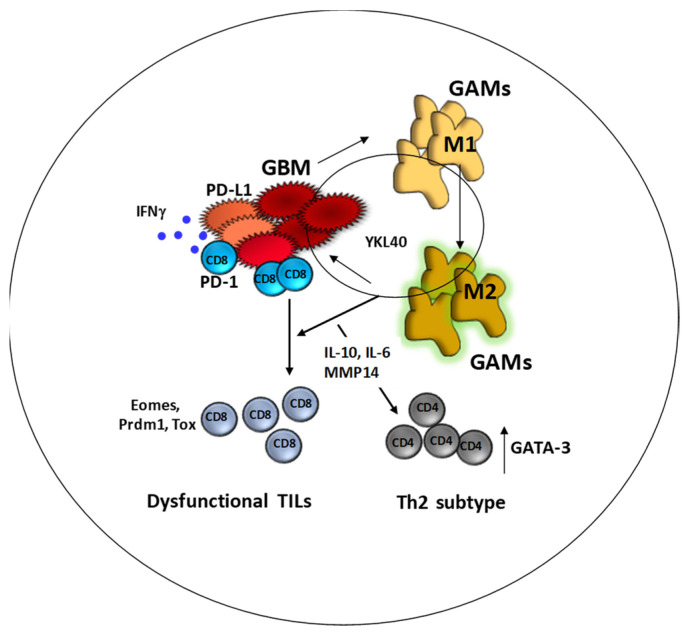
The presence of GAM within the microenvironment impact GBM proliferation, and reciprocally GBM cells impact on the infiltration of microglia and migration of macrophages. Within this context, GAMs are forced forwards M2 phenotypes by tumor cells. GBM cells can produce specific factors involved in M2 polarization. A recent mechanism involves the YKL40 production in inducing M2 polarization of GAM acquiring immunosuppressive and tumor supporting features. In this context, CD8+ effector TILs can recognize GBM cells. When activated, effector TILs produce IFNγ, previously described as involved in the PD-L1 overexpression of the surface of tumor cells. CD8+ TILs recognizing tumor cells are induced in a dysfunctional state and upregulate the markers related to a late dysfunction. In parallel, the M2-GAMs influence the shift of Th1 to Th2 subtype.

Therefore, we can reconstruct the mechanism through which the microenvironment can affect the dysfunction of T cells and consequently the responsiveness of patients treated to DC immunotherapy (Figure 3).

## 6. Conclusions

The recurrence of GBM is a major unmet medical need, mainly due to the lack of standard therapeutic approaches. Since checkpoint inhibitors in rGBM failed, much work has been conducted to elucidate the context of immune cells, one of the most critical components within the tumor microenvironment, in determining the potential failure or success of immunotherapeutic approaches. As a result, many signs of progress were achieved after the introduction of single-cell sequencing technologies, which allow in-depth and specific characterization of the main components responsible for such an immunosuppressive and hostile environment. Moreover, it is now evident that T cells can infiltrate rGBM and retain memory. However, it is crucial to envisage more specific studies directed to decipher the mechanisms that lead to their dysfunctional state.

As our data originated from such a small number of patients, these results require confirmation with more cases. However, the preliminary findings support the notion that deciphering the immune microenvironment with general genetic features can improve the success of immunotherapeutic approaches. Furthermore, we believe that combinatorial strategies more specifically targeted toward the microenvironment and the reprogramming of experienced T cells can enhance the responsiveness of rGBM.

## Supplementary Materials

The following are available online at https://www.mdpi.com/article/10.3390/cancers13246156/s1, Methods: RNA extraction and real-time PCR; Immunohistochemistry (IHC) and semiquantitative analysis of the immune infiltration. Table S1: Patient’s characterization. Table S2: Forward and reverse primers.
