# Supplementary material for: Deciphering the Labyrinthine System of the Immune Microenvironment in Recurrent Glioblastoma: Recent Original Advances and Lessons from Clinical Immunotherapeutic Approaches
Source: Cancers (Basel). 2021 Dec 7;13(24):6156. doi: 10.3390/cancers13246156 (PMC8699787; doi:10.3390/cancers13246156)
Supplement: Supplementary file 1 [file cancers-13-06156-s001.zip › cancers-1478862-supplementary.pdf]

Supplementary Data

Deciphering the labyrinthine system of the immune microen-vironment in recurrent glioblastoma: recent original advances and lessons from clinical immunotherapeutic approaches

Anghileri E., Patanè M., et al

Clinical characteristics

Patient #1, 59 years-old man, underwent surgery due to right parietal primary unmethylated pMGMT-GBM; then, he was treated with radiotherapy and chemotherapy as Stupp protocol. For local recurrence, after 16 months from the first surgery, he underwent to second gross total resection and enrolled in DENDR2 clinical study (NCT04002804): he recurred before starting immunotherapy (at 1.7 months from last surgery). Finally, he was treated with four doses of vaccines and recurred 2.3 months later, succumbing to further tumor progression after 7.4 months from the last surgery.

Patient #2, a 53 years-old woman, underwent her first surgery for MGMT unmethylated GBM located in the left parietal, occipital lobes; then, she was treated by Stupp protocol. After 16 months, the patient underwent a second surgery upon recurrence and was treated with an experimental drug with no response at a 2-month follow-up. At 20 months from the first operation, another surgery was done, followed by DENDR2 immunotherapy completed as for protocol. She died after 9.3 months from the last surgery due to recurrence.

Patient #3 had a diagnosis of histological grade II glioma diagnosed when she was 20 years old. Six years later, she underwent a second surgery for GBM, methylated in MGMT promoter and carrying the IDH1R132H mutation. Then she was treated with RT and TMZ as the standard of care with transitory disease stability. Six years later, GBM recurred, and the patient, after a second surgery,

was enrolled in the DENDR2 clinical study (NCT04002804) and treated with DC immunotherapy and TMZ as an adjuvant. Unfortunately, she succumbed to another recurrence of the tumor 25 months after her last surgery.

Patient #4 underwent her first surgery for MGMT methylated, IDH1H132S high-grade glioma when she was 37 years old and was treated by Stupp protocol. After 14 months, the patient underwent a second surgery upon recurrence and then was enrolled in the DENDR2 clinical study. She is still alive at 83 months from the second surgery.

Methods

RNA extraction and real-time PCR

Total RNA was extracted from frozen specimens using Trizol (ThermoFisher) and reverse-transcribed using the High-Capacity cDNA synthesis kit (ThermoFisher). The expression of genes was assessed by real-time PCR with the SYBRgreen method. Primers were defined using Primer Blast software (primers reported in Table S2). RNA from the commercially available normal brain (ThermoFisher) was used as a calibrator to calculate fold expression levels using the ΔΔCt method. The expression levels of target genes were normalized to the expression level of beta-actin.

Immunohistochemistry (IHC) and semiquantitative analysis of the immune infiltration

Paraffin-embedded 3 µm-thick sections were processed using AutostainerPlus (Dako, Agilent, Denmark), and antigen retrieval was performed in a PT Link pretreatment module (Dako, Agilent, Denmark) when requested. Briefly, slides were first blocked in 3% H2O2 (Sigma-Aldrich, USA), and then they were incubated with normal goat serum (Dako, Agilent, Denmark) and with the primary antibodies against the following: PD-L1 (1:100), CD3 (dilution 1:50), CD8 (dilution 1:50), and CD4 (dilution 1:50), (Dako, Agilent, Denmark), HLA-DR (dilution 1:50), CD163/MRQ-26 for microglia/macrophages (pre-diluted 1:2). Subsequently, sections were incubated with anti-mouse or anti-rabbit Envision conjugated with peroxidase-conjugated (Dako, Denmark) as a secondary antibody. Finally, slides were reacted with diaminobenzidine (DAB Substrate Chromogen System, Dako, Denmark) and counterstained with hematoxylin. Aperio Cs2 scanScope and ImageScope software were used for the semiquantitative analysis of staining levels.

Table S1. Patient's characterization

Characteristics					% Staining cells					
	Gender	IDH1	MGMT methylation	OS months	CD4	CD8	CD68	CD163	HLA-DR	PD-L1
pt #1	M	Wt	U	23.8	0	5	35	45	30	0
pt #2	F	Wt	U	29.2	0	5	35	55	45	3
pt #3	F	Mut IDH1H132S	M	25	15	55	50	75	70	20
pt #4	F	Mut IDH1R132H	M	>100	30	25	30	60	55	15

Table S2. Forward and reverse primers

Gene	Forward	Reverse
IL-6	CCAGAGCTGTGCAGATGAGT	GTTGGGTCAGGGGTGGTTAT
IL-10	TTGGGGCTTCCTAACTGCTA	TGGTTGGGGAATGAGGTTAG
MMP14	AGCATTGGGTGTTTGATGAGG	TCCAGAAGAGAGCAGCATCAA
IL-1B	CTCCAGGGACAGGATATGGA	CCCAAGGCCACAGGTATTTTG
CSF1R	AAAAGTCCTGACCCTCAACC	TCTACCACCCGGAAGAACAT
YKL40	GATGTGACGCTCTACGGCAT	CCCAAAGTTCCATCCTCCGA
TOX	TTACACTCACCCACC	ATTGCCTGGGTGACA
EOMES	ATGGGTGACCTGTGGCAAAG	TCCTGTCTCATCCAGTGGGA
BLIMP-1	GTGTGGTATTGTCGGGACTTTG	CAGTGCTCGGTTGCTTTAGAC
T-BET	CAAGGATTCCGGGAGAACT	TAGTGATCTCCCCCAAGGAA
GATA3	CGTTTTTCTGCCGTACCC	GGAAGCAACGTGAGCAAA
P2RY12	CCAGGAAAAAGGTGAACGTC	TGGCTCAGGGTGTAAGGAAT
TMEM119	GTACGTGATGCTGATTGCTGT	AAGGACGATGGGTAATAGGC
IFNG	CATCCAAGTGATGGCTGAAC	CTTCGACCTCGAAACAGCAT
PD1	TGGACTATGGGGAGCTGGATT	ACAATGGTGGCATACTCCGTC
CCL4	CCCAGCCAGCTGTGGTATTC	CATACACGTACTCCTGGACCC
CCL5	CTGCTGCTTTGCCTACATTG	GGTTCTTTCGGGTGACAAAG
beta-actin	GTCATTCCAAATATGAGATGCGTTG	TGTGGACTTGGGAGAGGACT
